# Actinomycin Analogs from Soil-Derived *Streptomyces* sp. PSU-S4-23 with Activity Against MRSA

**DOI:** 10.3390/life16010032

**Published:** 2025-12-25

**Authors:** Chollachai Klaysubun, Kamonnut Singkhamanan, Monwadee Wonglapsuwan, Sarunyou Chusri, Rattanaruji Pomwised, Komwit Surachat

**Affiliations:** 1 Department of Biomedical Sciences and Biomedical Engineering, Faculty of Medicine, Prince of Songkla University, Songkhla 90110, Thailand; chollachai951@gmail.com (C.K.);; 2 Division of Biological Science, Faculty of Science, Prince of Songkla University, Songkhla 90110, Thailand; 3 Division of Infectious Diseases, Department of Internal Medicine, Faculty of Medicine, Prince of Songkla University, Songkhla 90110, Thailand

**Keywords:** actinomycin X_2_, actinomycin D, actinomycin analogs, nonribosomal peptide synthetase (NRPS), biosynthetic gene clusters (BGCs), genome mining, LC–MS/MS profiling

## Abstract

Genome-based discovery provides a powerful approach for identifying bioactive natural products. In this study, *Streptomyces* sp. PSU-S4-23 was isolated from soil collected in southern Thailand. Genome analysis revealed a nonribosomal peptide synthetase (NRPS) biosynthetic gene cluster highly similar to the reference actinomycin D cluster, including canonical NRPS genes and a cytochrome P450 associated with oxidative tailoring. Genomic comparison indicated that this strain is distinct from its closest relative *S. caeni* CGMCC 4.7426^T^ with ANIb and dDDH values below the species delineation thresholds. In agar diffusion assays, the crude extract exhibited antibacterial activity against *Staphylococcus aureus* (MSSA and MRSA), *Bacillus subtilis*, *Bacillus cereus*, *Enterococcus faecalis*, *Staphylococcus epidermidis*, as well as inhibition of *Pseudomonas aeruginosa* and *Acinetobacter baumannii*. LC–MS/MS profiling of the crude ethyl-acetate extract was performed. GNPS feature-based molecular networking revealed ions corresponding to actinomycin X_2_ (*m*/*z* 1269.6), D (*m*/*z* 1255.6), and I (*m*/*z* 1271.6), confirming production of multiple actinomycin analogs. These findings highlight *Streptomyces* sp. PSU-S4-23 as a promising actinomycin-producing strain with potential relevance to antibiotic discovery.

## 1. Introduction

Antibiotics remain essential to modern medical care. However, the global rise in multidrug-resistant (MDR) bacteria poses a serious and escalating threat. Among these, methicillin-resistant *Staphylococcus aureus* (MRSA) continues to be one of the most common and challenging causes of antibiotic-resistant infections worldwide [1]. The spread of resistance across multiple drug classes has complicated the empirical treatment of *S. aureus* infections. This situation highlights a pressing need for novel antimicrobial agents.

Actinomycetes are widely recognized as a valuable source of alternative antibiotics [1]. Among this, *Streptomyces* stands out as the largest genus in the family Streptomycetaceae [2]. They are Gram-positive, saprotrophic bacteria that inhabit a range of environments, including soil and aquatic ecosystems. According to the List of Prokaryotic names with Standing in Nomenclature (LPSN), over 793 *Streptomyces* species have been validly published as of 25 October 2025. This genus is an exceptionally prolific producer of specialized metabolites. It accounts for the majority of known natural bioactive compounds, with an estimated 70–80% of clinically used antibiotics originating from *Streptomyces* species [3]. In addition to their antibacterial properties, many of these molecules exhibit strong antitumor activity. Approved cancer drugs such as doxorubicin, actinomycin, mitoxantrone, bleomycin, and mitomycin are all derived from *Streptomyces* metabolites [4]. Advances in genome sequencing have further revealed the vast biosynthetic potential of this genus. For example, *Streptomyces coelicolor* contains dozens of biosynthetic gene clusters (BGCs) for secondary metabolites. However, fewer than 10% are expressed under standard laboratory conditions [5]. This large reservoir of silent or poorly expressed BGCs highlights the immense untapped potential for discovering new therapeutics.

In the present study, we report the isolation of PSU-S4-23, a soil-derived strain capable of producing actinomycin. Its draft genome was sequenced and analyzed to identify antimicrobial biosynthetic gene clusters. Based on genome mining results, the strain was selected for metabolite production, antibacterial bioassays, and chemical analysis using LC–MS in combination with GNPS feature-based molecular networking.

## 2. Material and Methods

### 2.1. Isolation of Strain PSU-S4-23

Strain PSU-S4-23 was isolated from the top 5–10 cm layer of loamy soil collected from a area in the garden of the Faculty of Medicine, Prince of Songkla University, Hat Yai, Songkhla, Thailand (approximately 7°00′12.4″ N, 100°29′46.3″ E), on 15 November 2024. The soil sample was dried for 2 weeks at room temperature. For isolation, 1 g of dried soil was suspended in 9 mL of 0.85% NaCl. The obtained dilution (10^−3^–10^−1^ g/mL) liquid was plated on starch casein agar (SCA, Himedia, Mumbai, India) supplemented with nalidixic acid (25 µg/mL) and nystatin (50 µg/mL). After the plates were incubated at room temperature for 7–14 days, the single colony of strain PSU-S4-23 was transferred and purified on International *Streptomyces* Project medium 2 (ISP-2, Himedia, Mumbai, India) agar. Mycelia and spores were kept in glycerol solution (20%, *v*/*v*) at −20 °C.

### 2.2. Genome Sequencing, Assembly, and Annotation

Genomic DNA from PSU-S4-23 was extracted with the ZymoBIOMICS DNA Miniprep Kit (Zymo Research, Irvine, CA, USA) following the manufacturer’s instructions. DNA concentration and purity were measured using a Qubit4 fluorometer (Thermo Fisher Scientific, Waltham, MA, USA), and integrity was verified by agarose gel electrophoresis. Libraries were prepared with the Rapid Barcoding Kit 24 V14 (SQK-RBK114.24; Oxford Nanopore Technologies, Oxford, UK) and sequenced on an R10.4.1 flow cell using high-accuracy basecalling to generate long-read data. Quality-filtered reads were assembled with Flye v2.9.5 within the EPI2ME Labs environment [6]. The draft assembly was polished with Medaka v2.0.0, and assembly statistics were computed using QUAST v5.3 [7]. Genome completeness and contamination were estimated using BUSCO v5.8.0 [8] and CheckM v1.0.18 [9], respectively.

Functional annotation was performed with the NCBI Prokaryotic Genome Annotation Pipeline v6.10 [10] and the eggNOG-mapper v2.1.12 [11]. Additional functional assignments, including clusters of orthologous groups (COGs) and carbohydrate-active enzyme (CAZy) categories, were obtained with eggNOG-mapper v2.1.12. A circular genome map was generated with Proksee [12,13]. Antimicrobial resistance genes (ARGs), mobile genetic elements (MGEs), CRISPR–Cas loci, and prophage regions were identified using CARD v6.0.3 [14], MobileOG v1.6 [15], CRISPRCasFinder v4.2.20 [16], and PHASTEST v3.0 [17], respectively. Putative plasmid replicons were detected with PlasmidFinder v2.1 [18].

### 2.3. Phylogenetic Analysis

The 16S rRNA gene of strain PSU-S4-23 was extracted from the draft genome and initially queried against the EzBioCloud server [19]. For phylogenetic placement, 16S rRNA gene sequences of type strains from closely related *Streptomyces* species were retrieved from the NCBI GenBank database. Sequences were aligned with the ClustalW algorithm, and phylogenetic analyses were performed in MEGA v10 [20] using neighbor-joining [21], maximum-likelihood [22], and maximum-parsimony [23] methods. The maximum-likelihood and neighbor-joining results were calculated according to Kimura 2 parameter model and maximum-composite-likelihood model, respectively. Subtree-pruning- regrafting was used for the maximum-parsimony method. To achieve higher taxonomic resolution, multi-locus sequence analysis (MLSA) was conducted by uploading draft assemblies of strain PSU-S4-23 and related type strains to the Automated Multi-Locus Species Tree (autoMLST) pipeline v1.0 [24]. A complementary phylogenomic tree was generated with the Type Strain Genome Server (TYGS) [25]. Average Nucleotide Identity (ANI) was calculated using JSpeciesWS [26], and digital DNA–DNA hybridization (dDDH) values were obtained with the Genome-to-Genome Distance Calculator (GGDC) v2.1 [27] to evaluate genomic relatedness and species boundaries.

### 2.4. Identification and Netwoking of Putative BGCs

BGCs in the PSU-S4-23 genome were predicted with antiSMASH v8.0 [28]. Nonribosomal peptide synthetase (NRPS) and polyketide synthase (PKS) modules were examined for cluster-level features—including domain, adenylation/acyltransferase substrate predictions, and co-localized tailoring enzymes—to improve compound-level predictions. In addition, genes encoding bacteriocins were identified using BAGEL4 [29]. For comparative analysis with related *Streptomyces* strains, BGCs were organized into gene cluster families (GCFs) using BiG-SCAPE v2.0.0 [30] with the mix mode; clustering distance thresholds of 0.3, 0.4, and 0.5 were evaluated. Resulting similarity networks were visualized in Cytoscape v3.10.3 [31]. The -mibig option was enabled to annotate links to characterized clusters in the MIBiG 2.0 reference database [32].

### 2.5. Crude Extracts Preparation

A mycelial plug of PSU-S4-23 was used to inoculate 50 mL ISP-2 broth in a 250 mL Erlenmeyer flask. After 3 days of incubation at 28 °C with shaking at 180 rpm., 10 mL of the starter culture was transferred into 200 mL ISP-2 in a 500 mL Erlenmeyer flask and cultivated for 7 days under the same conditions (28 °C, 180 rpm.). The entire culture was extracted by vigorous shaking with an equal volume of ethyl acetate. Organic phases were combined and concentrated to dryness under reduced pressure using a rotary evaporator (Heidolph, Schwabach, Germany). The resulting crude extract was used for bioactivity assays.

### 2.6. Bioactivity Assays

Antibacterial activity of the crude extract was evaluated by the agar well-diffusion method [33] as a preliminary, semi-quantitative screen against *Staphylococcus aureus* ATCC 29213, methicillin-resistant *S. aureus* ATCC 43301, *Enterococcus faecalis* DMST 4736, *Staphylococcus epidermidis* ATCC 12228, *Bacillus subtilis* DMST 7988, *Bacillus cereus* DMST 11098, *Pseudomonas aeruginosa* DMST 4211, and *Acinetobacter baumannii* ATCC 19606. Briefly, 0.1 mL of each bacterial suspension (10^8^ CFU/mL) was spread onto Mueller–Hinton agar (MHA, HiMedia, Mumbai, India). Wells were created with a sterile cork borer. The crude extract (10 mg/mL in DMSO) was used as the test solution, and 50 μL was dispensed into each well. An equal volume of DMSO served as the negative control. All assays were performed in triplicate. Plates were incubated at 37 °C for 24 h, and zones of growth inhibition were measured and reported as mean ± standard deviation (SD).

### 2.7. LC-MS Analysis

The extract of strain PSU-S4-23 was analyzed on an Agilent 6545XT AdvanceBio LC/Q-TOF (Agilent Technologies, Santa Clara, CA, USA) operated in high-resolution mode. Samples were diluted in ethyl acetate, mixed 1:1 with ethanol containing sulfadimethoxine (100 ng/mL; internal standard), centrifuged at 14,000 rpm. for 10 min, and the supernatant transferred to LC vials. Chromatography used a Poroshell 120 EC-C18 column (2.1 × 100 mm, 2.7 µm) at 50 °C with mobile phase A (0.1% formic acid in water) and mobile phase B (0.1% formic acid in acetonitrile). A 10 µL sample was injected and eluted at a flow rate of 0.40 mL/min using the following gradient: 100% A for 0–0.5 min, then linearly decreased to 45% A/55% B at 10.5 min, followed by 25% A/75% B at 12.5 min. Solvent B was increased to 100% by 14.0 min and held until 17.0 min. The system was returned to 100% A at 17.5 min and equilibrated until 20.0 min. The mass spectrometer was operated in positive and negative electrospray ionization with reference-mass correction (positive: *m*/*z* 121.0509, 922.0098; negative: *m*/*z* 112.9856, 1033.9881), drying gas at 325 °C and 13 L/min, and sheath gas at 275 °C and 12 L/min. Raw data were processed in Agilent MassHunter Workstation v11.0. Feature-Based Molecular Networking (FBMN) was performed on the Global Natural Products Social (GNPS) platform [34,35,36] after preprocessing in MZmine v4.72 [37]. The data were filtered by removing all MS/MS fragment ions within ±17 Da of the precursor *m*/*z*, and by selecting the top 6 fragment ions within a ±50 Da window across the spectrum. Precursor and fragment ion tolerances were both set at 0.05 Da. Edges in the molecular network were kept if the cosine similarity score was ≥0.7 with at least 6 matched peaks. The resulting network was searched against GNPS spectral libraries for dereplication and annotation [38].

## 3. Results

### 3.1. Morphological and Genomic Characterization

**Figure 1:**
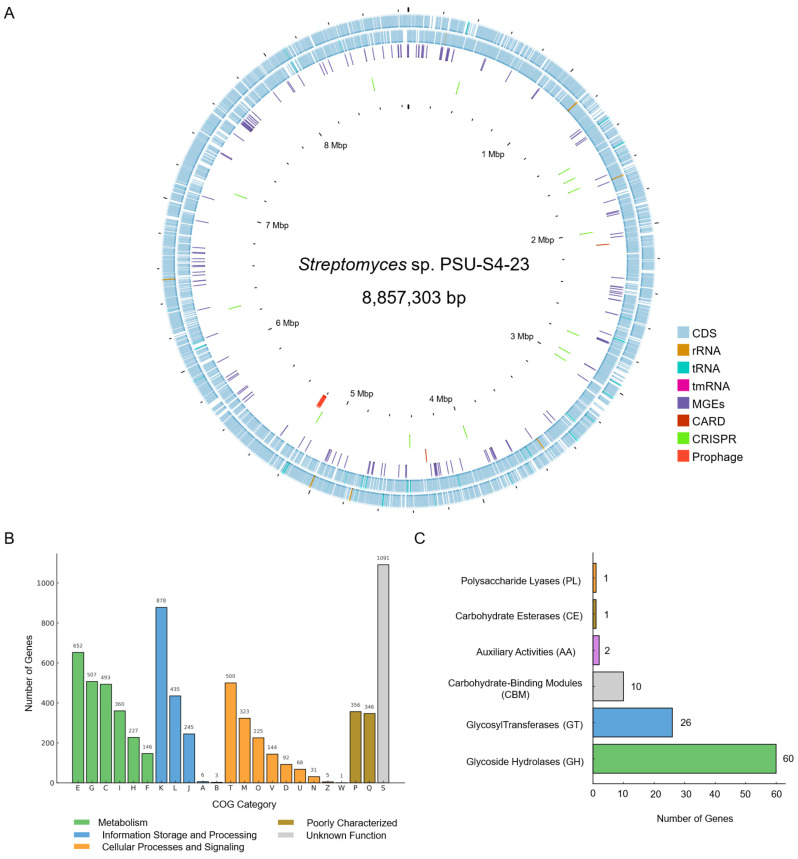
Genome overview and functional classification of *Streptomyces* sp. PSU-S4-23. (**A**) Circular genome map showing coding sequences, RNA genes, mobile genetic elements (MGEs), antibiotic-resistance genes (CARD), CRISPR loci, and prophages. (**B**) Distribution of genes assigned to Clusters of Orthologous Groups (COG) functional categories. (**C**) Classification of carbohydrate-active enzymes (CAZymes) into six major classes. For the COG one-letter codes are Amino acid transport and metabolism (E); Carbohydrate transport and metabolism (G); Energy production and conversion (C); Lipid transport and metabolism (I); Coenzyme transport and metabolism (H); Nucleotide transport and metabolism (F); Transcription (K); Replication, recombination, and repair (L); Translation, ribosomal structure, and biogenesis (J); RNA processing and modification (A); Chromatin structure and dynamics (B); Signal transduction mechanisms (T); Cell wall/membrane/envelope biogenesis (M); Posttranslational modification, protein turnover, chaperones (O); Defense mechanisms (V); Cell cycle control, cell division, chromosome partitioning (D); Intracellular trafficking, secretion, and vesicular transport (U); Cell Motility (N); Cytoskeleton (Z); Extracellular structures (W); Inorganic ion transport and metabolism (P); Secondary metabolites biosynthesis, transport, and catabolism (Q); Function unknown (S).

The macroscopic morphology of the PSU-S4-23 isolate is typical of *Streptomyces*, with a white color of the aerial mycelium and a pale-yellow vegetative mycelium, and it produced spores with yellow pigmentation on ISP-2 agar. The genome of this strain was sequenced to enable definitive taxonomic placement and to evaluate its biosynthetic capacity. The draft assembly comprises three contigs totaling 8,857,303 bp with an average G + C content of 71.8% (Figure 1A). Prokka annotation identified 7869 protein-coding sequences (CDSs), 87 tRNA genes, 19 rRNA genes (seven 16S rRNA, six 23S rRNA, and six 5S rRNA genes), and one tmRNA gene. Assembly quality metrics indicated an N50 of 8,704,871 bp with no gaps, 99.7% complete BUSCOs using the Streptomycetales lineage, and 100.0% completeness with 2.22% contamination according to CheckM (Streptomycetaceae marker lineage), supporting a highly complete, low-contamination draft genome.

Genome screening with CARD identified two strict hits in strain PSU-S4-23, including a vanH-like reductase (38.5% identity to reference), consistent with a divergent vanH homolog implicated in glycopeptide (vancomycin) resistance via target modification, and a rifampin monooxygenase (rox) (73.4% identity), indicating a rifamycin-inactivation determinant that can act on rifampin, rifaximin, and rifapentine. These findings suggest intrinsic genomic potential for resistance to glycopeptides and rifamycins, although phenotypic resistance remains to be confirmed experimentally. CRISPRCasFinder identified fourteen putative CRISPR arrays in the strain PSU-S4-23 genome. No cas loci were predicted. PHASTEST detected a single intact prophage (*Streptomyces* phages PapayaSalad), indicating a history of phage interaction that may contribute to genome plasticity and secondary metabolite diversification in strain PSU-S4-23. Moreover, no plasmid replicon was detected in this strain.

COG classification shows that the most abundant categories are K (Transcription), E (Amino acid transport and metabolism), G (Carbohydrate transport and metabolism), T (Signal transduction mechanisms) and C (Energy production and conversion) (Figure 1B), reflecting a soil lifestyle requiring versatile substrate utilization and tight regulatory control. CAZy annotation identified glycoside hydrolase (GH = 60 genes), glycoside hydrolase (GT = 26 genes), carbohydrate-binding module (CBM = 10 genes), auxiliary activities (AA = 2 genes), carbohydrate esterase (CE = 1 gene), and polysaccharide lyase (PL = 1 gene). The GH complement spans degradation and remodeling of cellulose/hemicellulose, storage glucans, and chitin, including GH6 and GH12 (cellulases), GH5 and GH26 (cellulases/mannanases), GH43 (α-L-arabinofuranosidases), GH31/GH30 (exo-α/β-glucosidases), GH32 (fructan/fructosidase), GH13 (α-amylases, 4-α-glucanotransferases, and branching enzymes), GH20 (β-hexosaminidases), and GH88 (unsaturated glucuronyl hydrolases). Collectively, these features point to a capacity for environmental polysaccharide turnover and glycosidic-bond construction, consistent with saprotrophic ecology and its genomic potential for complex natural-product biosynthesis (Figure 1C).

### 3.2. Taxonomic Assignment

**Figure 2:**
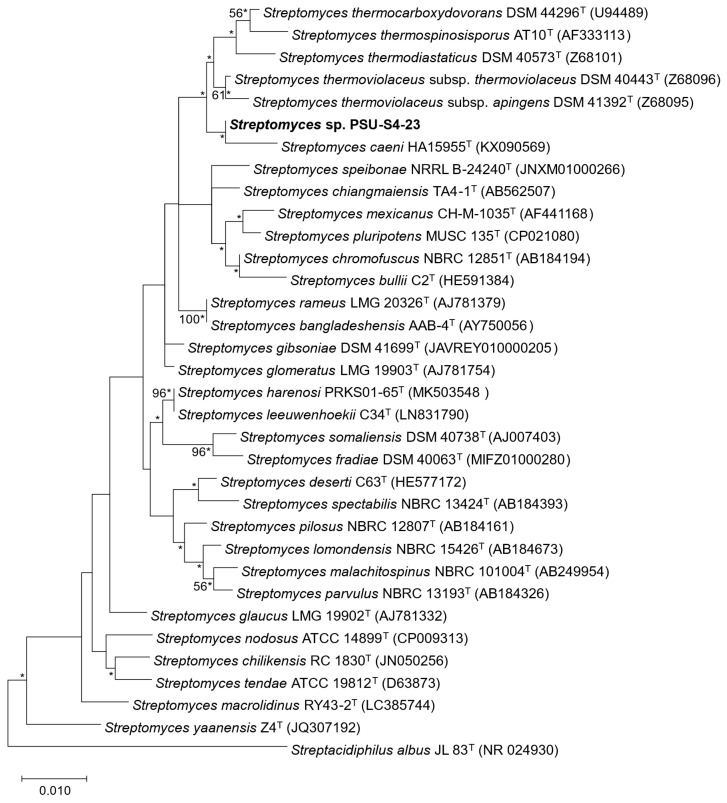
Maximum-likelihood phylogeny of the 16S rRNA gene showing the placement of strain PSU-S4-23 within the genus *Streptomyces*. *Streptacidiphilus albus* JL 83^T^ serves as the outgroup. Node values are ML bootstrap percentages (1000 replicates); values > 50% are shown. Bar, 0.01 substitutions per nucleotide position. Asterisks indicate that the corresponding branches were also recovered in trees generated with the neighbor-joining and maximum-parsimony methods.

The complete 16S rRNA gene of strain PSU-S4-23 was retrieved from the genome assembly. Comparative analyses using EzBioCloud and NCBI GenBank indicated highest similarity to *Streptomyces thermoviolaceus* subsp. *thermoviolaceus* DSM 40443^T^ (99.10%), *Streptomyces glomeratus* LMG 19903^T^ (98.96%), *Streptomyces mexicanus* CH-M-1035^T^ (98.83%), and *Streptomyces caeni* HA15955^T^ (98.83%). A maximum-likelihood tree based on the 16S rRNA gene placed strain PSU-S4-23 within the genus *Streptomyces* (Figure 2, Figures S1 and S2), forming a well-supported cluster with *S. caeni* HA15955^T^, whereas *S. thermoviolaceus* subsp. *thermoviolaceus* DSM 40443^T^, *S. glomeratus* LMG 19903^T^, and *S. mexicanus* CH-M-1035^T^ grouped on a separate branch. Given the high 16S rRNA sequence conservation typical of *Streptomyces*, whole-genome comparisons are required to refine the taxonomic placement of *Streptomyces* sp. PSU-S4-23.

**Figure 3:**
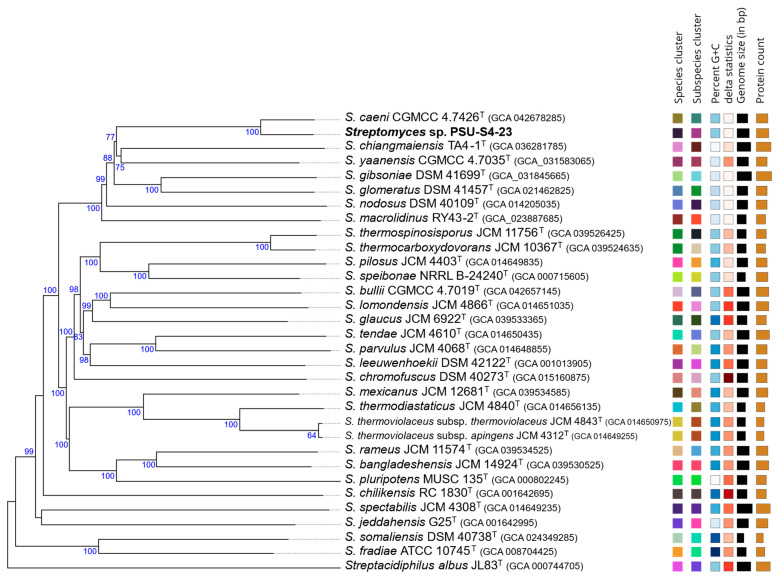
Phylogenomic placement of strain PSU-S4-23 among related *Streptomyces* species. Tree inferred with FastME 2.1.6.1 from GBDP distances calculated from genome sequences. The branch lengths are scaled in terms of GBDP distance formula d5; numbers above branches are GBDP pseudo-bootstrap support values > 60% from 100 replications, with an average branch support of 93.0%.

Multilocus sequence analysis inferred with autoMLST showed that *Streptomyces* sp. PSU-S4-23 forms a monophyletic clade with *Streptomyces caeni* CGMCC 4.7426^T^ (Figure S3), consistent with the whole-genome phylogeny inferred using TYGS (Figure 3). Average nucleotide identity based on BLAST v2.2.29+ (ANIb) was then calculated with JSpeciesWS (Figure S4), and digital DNA–DNA hybridization (dDDH) was estimated with the GGDC (Figure S5) for *Streptomyces* sp. PSU-S4-23 and closely related type strains. *Streptomyces* sp. PSU-S4-23 shares less than 95% ANI and less than 70% dDDH with all closely related type strains, including its closest relative *S. caeni* CGMCC 4.7426^T^ (ANIb = 94.69%; dDDH = 68.2%). As these values fall below the accepted species thresholds (95–96% ANI and 70% dDDH), *Streptomyces* sp. PSU-S4-23 should be considered a distinct species within the genus *Streptomyces*.

### 3.3. Analysis of BGCs

The *Streptomyces* sp. PSU-S4-23 genome was analyzed with antiSMASH v8.0 to identify putative BGCs, revealing 24 complete clusters. Six BGCs showed high-confidence similarity to characterized references ectoine, desferrioxamine B/E, albaflavenone, geosmin, actinomycin D, and hopene (Table 1). Additional clusters were provisionally assigned to known families with lower confidence, including aborycin, azodyrecin A/B/C, tripartilactam/niizalactam C, spore pigment, echinomycin, carotenoid, 14-hydroxyisochainin, largimycin A1/A2/A3, grincamycin, galtamycin C/D, and informatipeptin. BAGEL4 further detected a Zoocin A–like bacteriocin locus. Across the genome, PKS clusters were most abundant, followed by terpene and NRPS classes. Collectively, the annotated BGCs point to a broad capacity for the biosynthesis of antibiotics and other bioactive metabolites.

**Figure 4:**
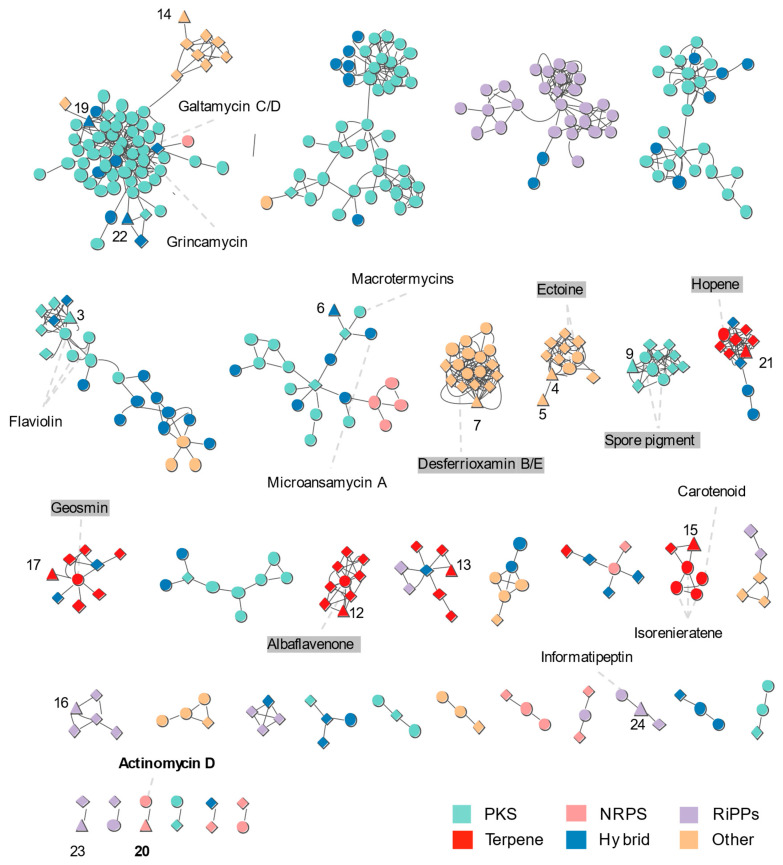
Sequence similarity network of the biosynthetic gene clusters detected in *Streptomyces* sp. PSU-S4-23 compared with BGCs in the MIBiG database and closely related *Streptomyces* strains. The numbers indicate the order of BGCs predicted by antiSMASH in the PSU-S4-23 genome. Triangular nodes represent BGCs from this strain, and circular nodes represent MIBiG BGCs and diamond nodes represent closely *Streptomyces* BGCs. Singletons are excluded from the figure. Gray dashed lines are annotated compound name to its corresponding BGC cluster in the PSU-S4-23 genome. Colors were schemed according to different BGC category annotations. Grey labels highlight selected gene cluster families that represent conserved core BGCs shared among all closely related *Streptomyces* strains.

To explore BGC diversity, we constructed a BiG-SCAPE sequence-similarity network from the BGCs of *Streptomyces* sp. PSU-S4-23, seven closely related *Streptomyces* strains, and MIBiG references, resolving the dataset into multiple gene-cluster families (GCFs) (Figure 4). antiSMASH identified 463 BGC-containing regions across *Streptomyces* sp. PSU-S4-23 and the seven relatives *S. caeni* CGMCC 4.7426^T^, *S. chiangmaiensis* TA4-1^T^, *S. yaanensis* CGMCC 4.7035^T^, *S. gibsoniae* DSM 41699^T^, *S. glomeratus* DSM 41457^T^, *S. nodosus* DSM 40109^T^, and *S. macrolidinus* RY43-2^T^. These regions were clustered by BiG-SCAPE into 164 GCFs and assembled into a sequence-similarity network. NRPS, terpene, siderophore, and ectoine classes formed the largest interconnected components, whereas 93 regions remained as singletons.

*Streptomyces* sp. PSU-S4-23 shares conserved GCFs with the seven closely related strains, including terpene pathways (geosmin, hopene, albaflavenone), ectoine, the NI-type siderophore family related to desferrioxamine, and the spore-pigment type II PKS (T2PKS), reflecting core ecological functions (Figure 4). In these groups, *Streptomyces* sp. PSU-S4-23 nodes (triangles) mix with nodes from closely related strains (diamonds), indicating high conservation of the BGCs. By contrast, the actinomycin NRPS (Cluster 20) forms a GCF connected to the MIBiG actinomycin D reference and is absent from closely related strains. *Streptomyces* sp. PSU-S4-23 is further linked to PKS families such as flaviolin (Cluster 3), grincamycin (Cluster 19), and galtamycin C/D (Cluster 22), with only a subset of the other genomes contributing nodes to these clusters. Moreover, some GCFs associated with PSU-S4-23 nodes such as carotenoid informatipeptin and macrotetromycins or microansamycin-like clusters contain few nodes from other genomes. Overall, the network indicates that *Streptomyces* sp. PSU-S4-23 retains core ecological BGCs shared with its relatives, while also possessing several uncommon NRPS and PKS families that may encode unique metabolites.

**Figure 5:**
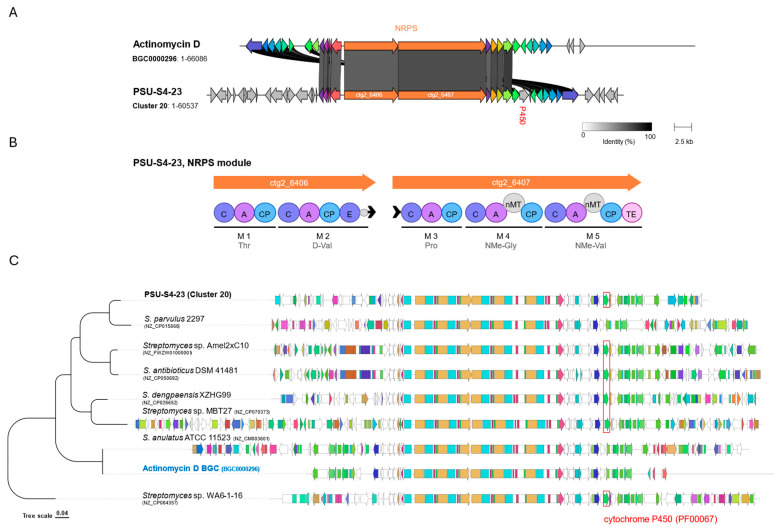
Comparative analysis of the actinomycin-like NRPS cluster from *Streptomyces* sp. PSU-S4-23 (Cluster 20) against the reference actinomycin D BGC (BGC0000296). (**A**) Synteny comparison between BGC0000296 and actinomycin-like cluster from *Streptomyces* sp. PSU-S4-23. The NRPS backbone (orange arrows) is highlighted; grayscale ribbons indicate pairwise gene identity, and the in-cluster cytochrome P450 (red label) is marked. (**B**) NRPS domain in *Streptomyces* sp. PSU-S4-23 consistent with a pentapeptide actinomycin scaffold. (**C**) Actinomycin-like clusters comparison across multiple *Streptomyces* genomes. The boxed region highlights a conserved P450 (PF00067) among actinomycin-like clusters; the actinomycin D (BGC0000296) is shown as a reference. Abbreviations: C, condensation; A, adenylation; CP/PCP, peptidyl carrier protein (T); E, epimerization; nMT, N-methyltransferase; TE, thioesterase.

Recent studies show that actinomycins I (X_0β_) and X_2_ form via sequential oxidation at the γ-carbon of the prolyl residue catalyzed by a cytochrome P450 enzyme [39,40]. In *Streptomyces* sp. PSU-S4-23, an NRPS biosynthetic gene cluster (Cluster 20) was identified that closely matches the actinomycin D reference BGC0000296 (Figure 5). The locus comprises 41 open reading frames (ORFs) (Figure 5A), including two ORFs (ctg2_6406/ctg2_6407) encoding five-module NRPSs whose domains predict the canonical pentapeptide: M1 Thr, M2 D-Val (epimerization domain present), M3 Pro, M4 N-methyl-Gly (nMT), and M5 N-methyl-Val (nMT) (Figure 5B). Strong module collinearity, together with 20 best-hit ortholog pairs (≥30% amino-acid identity) (Table S1) and conserved flanking tailoring/transport genes, supports annotation of cluster 20 as actinomycin-like. Comparative analysis of actinomycin-type clusters from PSU-S4-23 and closely related *Streptomyces* strains, displayed together with a tree showing their relationships based on BGC similarity (Figure 5C). A conserved cytochrome P450 gene block (highlighted in red at the right end of the cluster) is present in PSU-S4-23 and in other known actinomycin producers. This conserved P450 region supports that the PSU-S4-23 actinomycin BGC is functionally capable of introducing oxidative modifications that generate oxidized actinomycin analogs such as actinomycin X_2_ and I.

### 3.4. Antimicrobial-Activity Testing

**Figure 6:**
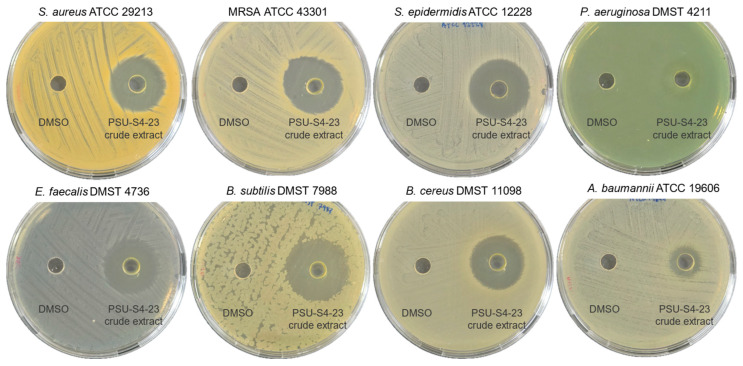
Antibacterial activity of the *Streptomyces* sp. PSU-S4-23 crude extract in agar well assays.

Guided by the genome analysis, *Streptomyces* sp. PSU-S4-23 was evaluated for antibiotic production under laboratory conditions. Cultures were grown for 7 days in ISP-2 medium, and the clarified supernatant was extracted with ethyl acetate to yield a yellow crude extract. Antibacterial activity was assessed by agar well diffusion with DMSO as the negative control (no solvent-associated inhibition; Figure 6). The extract inhibited multiple Gram-positive bacteria *S. aureus* (18.7 ± 0.6 mm), methicillin-resistant *S. aureus* (20.3 ± 0.6 mm), *B. subtilis* (21.7 ± 0.6 mm), *B. cereus* (20.3 ± 0.6 mm), *E. faecalis* (20.7 ± 0.6 mm) and *S. epidermidis* (20.7 ± 0.6 mm), and also showed activity against Gram-negative pathogens *P. aeruginosa* (13.0 ± 1.0 mm) and *A. baumannii* (10.7 ± 0.6 mm). The observed pattern, larger zones for Gram-positive and smaller for Gram-negative species, is consistent with outer-membrane–mediated tolerance in Gram-negative bacteria. These results indicate that the crude extract of *Streptomyces* sp. PSU-S4-23 contains extracellular metabolites with broad-spectrum antibacterial activity, particularly strong effects against Gram-positive pathogens such as MRSA.

### 3.5. LC–MS/MS Profiling and Feature-Based Molecular Networking (GNPS)

**Figure 7:**
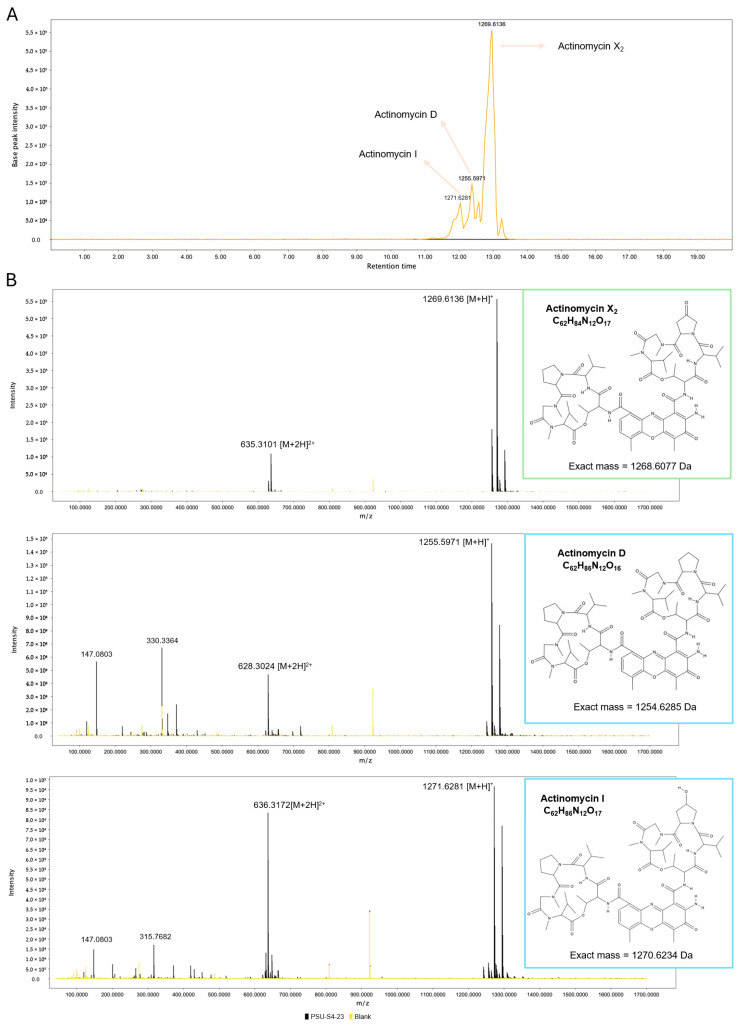
LC–MS analysis of actinomycin analogs in the *Streptomyces* sp. PSU-S4-23 extract. (**A**) The base-peak chromatogram shows three peaks based on *m*/*z* values for [M+H]^+^ are assigned to actinomycin D, actinomycin I, and the dominant actinomycin X_2_. (**B**) Full-scan MS^1^ spectra at the apex of each peak show the corresponding [M+H]^+^ (and characteristic [M+2H]^2+^) ions; chemical structures are shown in the insets. Traces are overlaid: black, *Streptomyces* sp. PSU-S4-23 extract; yellow, solvent blank.

LC–MS profiling of the *Streptomyces* sp. PSU-S4-23 crude extract was conducted in positive-ion mode, which yielded the most intense and interpretable signals. The resulting base-peak chromatogram revealed a narrow cluster of peaks between 12.0 and 13.0 min (Figure 7A). Within this window, several parent ions diagnostic of actinomycins were detected. These included *m*/*z* 1255.6 [M+H]^+^ (actinomycin D), *m*/*z* 1269.6 [M+H]^+^ (actinomycin X_2_; +14 Da compared to D), and *m*/*z* 1271.6 [M+H]^+^ (actinomycin I; +16 Da compared to D). Expected doubly charged ions were also present in the *m*/*z* 628–636 range [M+2H]^2+^ (Figure 7B). FBMN of the LC–MS/MS data grouped these ions into a single molecular family. Nodes were observed at *m*/*z* 628.3208, 635.3102, and 636.3183, corresponding to the [M+2H]^2+^ species (Figure S6). The observed *m*/*z* differences of +7 and +8 align with the +14 and +16 Da mass shifts between actinomycin D, X_2_, and I at a unit charge (D → X2 → I). The node at *m*/*z* 628.3208 matched the calculated [M+2H]^2+^ for actinomycin D, confirming identity of the compound. Collectively, the chromatographic retention times, accurate mass measurements, and molecular network topology support the presence of a mixture of actinomycins D, X_2_, and I. Notably, the dominant peak at 12.7–13.0 min (*m*/*z* 1269.6135 [M+H]^+^, C_62_H_85_N_12_O_17_) identified actinomycin X_2_ as the predominant analog detected, with D and I present as minor co-metabolites. These results are consistent with the identified NRPS/P450 biosynthetic gene cluster and align with the observed antibacterial activity in bioassays.

## 4. Discussion

The genus *Streptomyces* (phylum Actinomycetota) is one of the most prolific sources of biologically active natural products in modern medicine. In this study, we isolated *Streptomyces* sp. PSU-S4-23 from a soil sample. Genome-based analysis confirmed its placement within the *Streptomyces* genus. Its closest related strain was identified as *S. caeni* CGMCC 4.7426^T^. However, dDDH (68.2%) and ANIb (94.69%) were both below the species-level thresholds [41]. Whole-genome phylogeny generated by TYGS supported this placement and further reinforced its taxonomic separation [42]. Although genomic evidence indicates *Streptomyces* sp. PSU-S4-23 may represent a novel species, formal classification will require a polyphasic taxonomic approach.

Functional annotation revealed that several COG categories were enriched. These included genes involved in transcription, amino acid transport and metabolism, carbohydrate metabolism, signal transduction, and energy production. The CAZy profile of *Streptomyces* sp. PSU-S4-23 also indicated a diverse repertoire of enzymes. These comprised glycoside hydrolases, glycosyltransferases, polysaccharide lyases, and carbohydrate-binding modules. This enzymatic profile aligns with traits of soil-dwelling saprotrophs and supports roles in cell-wall remodeling and stress response. In resource-limited and competitive environments, microorganisms must efficiently access nutrients to survive. This ecological pressure often drives the activation of secondary metabolic pathways [43,44]. The overall biosynthetic capacity of *Streptomyces* is ecologically justified by its saprotrophic genomic signature, where secondary metabolites serve as tools for competition and defense.

Comparative BGC analysis further highlights the unique biosynthetic potential of *Streptomyces* sp. PSU-S4-23. In the BiG-SCAPE sequence-similarity network, this strain shared conserved GCFs with seven closely related *Streptomyces* genomes. These shared pathways included biosynthesis of geosmin, hopene, carotenoids, ectoine, desferrioxamine-like siderophores, and type II and III PKSs. Such features reflect core ecological functions. In contrast, *Streptomyces* sp. PSU-S4-23 also contributed unique nodes to sparsely populated GCFs. Notably, it harbored an actinomycin NRPS cluster with strong similarity to the MIBiG reference for actinomycin D (BGC0000296). Structurally, actinomycins are composed of a 2-aminophenoxazin-3-one chromophore linked to two cyclic pentapeptide lactone rings. Their biosynthesis typically spans 50 kb and involves around 28 genes, as seen in *Streptomyces anulatus* [45,46]. The actinomycin D gene cluster contains three key genes—*acmA*, *acmB*, and *acmC*—which activate amino acids and construct the peptide backbone. A post-assembly oxidative tailoring step converts actinomycin D into “X-type” variants. This reaction is catalyzed by the *acmM* gene product, a cytochrome P450 monooxygenase [39]. In *S. antibioticus* (a producer of actinomycins X_2_ and I), cytochrome P450 monooxygenase mediates sequential proline hydroxylation and oxidation, forming actinomycin I and then X_2_ [47,48]. Interestingly, while closely related *Streptomyces* strains lack this type of NRPS/P450 cluster, *Streptomyces* sp. PSU-S4-23 encodes a complete actinomycin pathway. A comprehensive review of *Streptomyces* species confirmed to harbor the actinomycin BGC or produce actinomycin analogs. These include isolates from diverse habitats, ranging from terrestrial soils to marine and Antarctic environments. Examples include *S. chrysomallus* [46], *S. parvulus* [49], *S. antibioticus* [48], *S. griseoruber* [50], *S. iakyrus* [51] and *S. lannensis* [52]. Other sources include mangrove-derived strains like *S. costaricanus* and *S. smyrnaeus* [45,53], marine isolates such as *S. heliomycini* [54], and Antarctic strains like *S. fildesensis* [55]. These producers generate a variety of actinomycin analogs. Many of these compounds exhibit structural modifications—such as halogenation or hydroxylation—that enhance their bioactivity. Collectively, genomic investigations underscore that actinomycin biosynthesis is not confined to a single lineage. It occurs across diverse ecological and genetic contexts within the *Streptomyces* genus.

Bioactivity assays revealed antibacterial effects. The ethyl-acetate extract of PSU-S4-23 inhibited a panel of pathogens in agar diffusion assays. Notably large zones of inhibition were observed against Gram-positive bacteria such as *Staphylococcus aureus* (including MRSA), *Bacillus subtilis*, *B. cereus*, *Enterococcus faecalis*, and *Staphylococcus epidermidis*. The extract also showed activity against Gram-negative strains such as *Pseudomonas aeruginosa* and *Acinetobacter baumannii*. This partial activity reflects the outer membrane barrier typical of Gram-negative bacteria, which limits antibiotic permeability. Several *Streptomyces* strains producing actinomycin mixtures have shown similar bioactivity profiles. Extracts from *S. griseoruber* Py2 (rich in actinomycin D) inhibit *E. coli* [50]. Likewise, *S. parvulus* Av-R5, which produces actinomycin D and X_0β_, displays strong activity against *Klebsiella pneumoniae* and *P. aeruginosa* [56]. Another strain, M7, produces a blend of actinomycins V, X_2_, and D. Purified compounds from M7 demonstrated potent effects against MRSA, vancomycin-resistant Enterococcus (VRE), *K. pneumoniae*, and *E. coli*. Among these, actinomycin X_2_ consistently showed superior activity compared to D and V [57].

Metabolite profiling confirmed the presence of actinomycin analogs. LC–MS analysis revealed a dominant feature at *m*/*z* 1269.6 [M+H]^+^, with a retention time of 12.7–13.0 min. This signal corresponds to actinomycin X_2_ (C_62_H_84_N_12_O_17_, exact mass = 1268.6077 Da). Additional peaks indicated the presence of actinomycins D and I. These results align with other recent findings. For instance, *Streptomyces* sp. DH7, a known actinomycin D producer, displayed potent activity against MRSA [58]. Similarly, *S. griseoruber* NBRC 12873 and *S. heliomycini* from marine environments were found to produce X_2_, D, and I analogs with strong antibacterial effects [50,54]. Mechanistically, actinomycin D binds tightly to DNA by intercalating between guanine–cytosine base pairs. This blocks transcription by RNA polymerase, ultimately halting gene expression [59]. The ability of actinomycins to disrupt both bacterial and tumor cell growth underlines their clinical relevance [54,57]. The agar diffusion data in this study offer preliminary confirmation of the extract’s bioactivity. To better assess clinical utility, future studies should focus on determining the MIC and MBC values of purified compounds. Since many actinomycins are known to exhibit cytotoxic effects, it is also important to evaluate the safety profile of these compounds. Cytotoxicity screening of the purified compounds will be necessary to establish their therapeutic window. These evaluations will help determine whether the actinomycin analogs from *Streptomyces* sp. PSU-S4-23 are viable candidates for development as selective antibacterial agents. In conclusion, the ethyl-acetate extract of *Streptomyces* sp. PSU-S4-23 produces actinomycins X_2_, D, and I, with X_2_ as the predominant metabolite. Its crude extract exhibited antibacterial activity against a broad panel of pathogens, including multidrug-resistant strains. Together, the genomic, metabolic, and bioactivity data support *Streptomyces* sp. PSU-S4-23 as a promising actinomycin-producing isolate.

## 5. Conclusions

This study highlights the potential of *Streptomyces* sp. PSU-S4-23 as a novel source of biologically active actinomycin analogs. Genomic analysis revealed a complete actinomycin NRPS gene cluster located adjacent to a cytochrome P450 enzyme. The crude ethyl-acetate extract exhibited strong antibacterial activity, particularly against MRSA. LC–MS profiling, supported by molecular networking, confirmed the production of multiple actinomycins. Among these, actinomycin X_2_ was the predominant analog, followed by actinomycins D and I. This is the first report of an actinomycin-producing *Streptomyces* lineage closely related to *S. caeni*. This lineage has been genomically characterized and linked to a defined actinomycin biosynthetic gene cluster and anti-MRSA activity. These combined findings position *Streptomyces* sp. PSU-S4-23 as a promising candidate for future development of actinomycin-based treatments targeting multidrug-resistant *S. aureus* infections.

## Figures and Tables

**Table 1:** BGCs in *Streptomyces* sp. PSU-S4-23 predicted by AntiSMASH v.8.0.

Cluster	Size (bp)	BGC Type	Most Similar Known Cluster	Similarity Confidence	MIBiG Accession
1	51,854	T1PKS, hglE-KS	–	–	–
2	21,056	Terpene	Aborycin	Low	BGC0002285
3	41,065	T3PKS	–	–	–
4	10,399	Ectoine	Ectoine	High	BGC0000853
5	22,601	Indole	Azodyrecin A/B/C	Low	BGC0002805
6	176,542	NRPS-like, butyrolactone, T1PKS, NRPS	Tripartilactam/ niizalactam C	Medium	BGC0002517
7	29,773	NI-siderophore	Desferrioxamin B/E	High	BGC0000940
8	26,458	Lanthipeptide-class-i	–	–	–
9	72,504	T2PKS	Spore pigment	Medium	BGC0000271
10	41,761	NRPS-like	Echinomycin	Low	BGC0000339
11	12,829	Hydrogen-cyanide	–		
12	21,056	Terpene	Albaflavenone	High	BGC0000660
13	21,125	Terpene-precursor	–	–	–
14	29,959	NI-siderophore	–	–	–
15	25,271	Terpene	Carotenoid	Medium	BGC0000633
16	11,341	RiPP-like	14-hydroxyisochainin	Low	BGC0002788
17	22,172	Terpene	Geosmin	High	BGC0001181
18	84,785	transAT-PKS, NRPS, T1PKS, PKS-like	Largimycin A1/A2/A3	Low	BGC0001853
19	78,990	NI-siderophore, T2PKS, butyrolactone	Grincamycin	Low	BGC0000229
20	60,537	NRPS	Actinomycin D	High	BGC0000296
21	26,763	Terpene	Hopene	High	BGC0000663
22	43,072	T1PKS, butyrolactone	Galtamycin C/D	Low	BGC0002140
23	22,787	Redox-cofactor	–	–	–
24	10,216	RiPP-like	Informatipeptin	Low	BGC0000518

## Data Availability

The molecular networking results can be publicly accessed at: https://gnps.ucsd.edu/ProteoSAFe/status.jsp?task=f70e25f4fc3f4f149d6037888f89f7be (accessed on 1 October 2025). The draft genome of the PSU-S4-23 was deposited at NCBI GenBank under BioProject accession number PRJNA1346719.

## Supplementary Materials

The following supporting information can be downloaded at https://www.mdpi.com/article/10.3390/life16010032/s1, Figure S1: Neighbor-joining tree based on 16S rRNA gene sequences showing the relationship between strain PSU-S4-23 and other closely related *Streptomyces*; Figure S2: Maximum parsimony phylogenetic tree based on 16S rRNA gene sequences showing the relationship between strain PSU-S4-23 and closely related *Streptomyces*; Figure S3: Phylogenomic tree generated by autoMLST showing the relationship of strain PSU-S4-23 to related species within the genus *Streptomyces*; Figure S4: Average Nucleotide Identity (ANI) heatmap and clustering of *Streptomyces* genomes; Figure S5: Digital DNA–DNA hybridization (dDDH) heatmap among *Streptomyces* genomes; Figure S6: Actinomycin D–associated cluster in the GNPS molecular network of the ethyl-acetate extract from *Streptomyces* sp. PSU-S4-23; Table S1: Assembly statistics and quality metrics for the draft genome of *Streptomyces* sp. PSU-S4-23; Table S2: Comparative analysis of the BGC 20 in PSU-S4-23 and the actinomycin D cluster (BGC0000296). The molecular networking job can be publicly accessed at https://gnps.ucsd.edu/ProteoSAFe/status.jsp?task=f70e25f4fc3f4f149d6037888f89f7be (accessed on 1 October 2025).
