# Supplementary material for: Actinomycin Analogs from Soil-Derived Streptomyces sp. PSU-S4-23 with Activity Against MRSA
Source: Life (Basel). 2025 Dec 25;16(1):32. doi: 10.3390/life16010032 (PMC12843237; doi:10.3390/life16010032)
Supplement: Supplementary file 1 [file life-16-00032-s001.zip › life-4019222-supplementary.pdf]

Supplementary data

Actinomycin analogs from soil-derived Streptomyces sp. PSU-S4-23 with activity against MRSA

Figure S1. Neighbour-joining tree based on 16S rRNA gene sequences showing the relationship between strain PSU-S4-23 and other closely related *Streptomyces*. All positions containing gaps and missing data were eliminated. There were a total of 1389 positions in the final dataset. Bootstrap values (>50 %) based on 1000 replicates are shown at branch nodes. *Streptacidiphilus albus* JL 83^T was used as the outgroup. Bar, 0.005 nucleotide substitution rate (Knuc) units.

Figure S2. Maximum parsimony phylogenetic tree based on 16S rRNA gene sequences showing the relationship between strain PSU-S4-23 and closely related *Streptomyces*. All positions containing gaps and missing data were eliminated. There were a total of 1389 positions in the final dataset. *Streptacidiphilus albus* JL 83^T was used as an outgroup. Bootstrap values 50 % (based on 1000 replications) are shown at branch points. Bar, 0.005 substitutions per nucleotide position.

Figure S3. Phylogenomic tree generated by autoMLST showing the relationship of strain PSU-S4-23 to related species within the genus *Streptomyces*.

Figure S4. Average Nucleotide Identity (ANI) heatmap and clustering of *Streptomyces* genomes.

Figure S5. Digital DNA–DNA hybridization (dDDH) heatmap among *Streptomyces* genomes.

Figure S6. Actinomycin D–associated cluster in the GNPS molecular network of the ethyl-acetate extract from *Streptomyces* sp. PSU-S4-23.

Table S1. Assembly statistics and quality metrics for the draft genome of *Streptomyces* sp. PSU-S4-23.

Table S2. Comparative analysis of the BGC Cluster 20 in PSU-S4-23 and the actinomycin D cluster (BGC0000296).

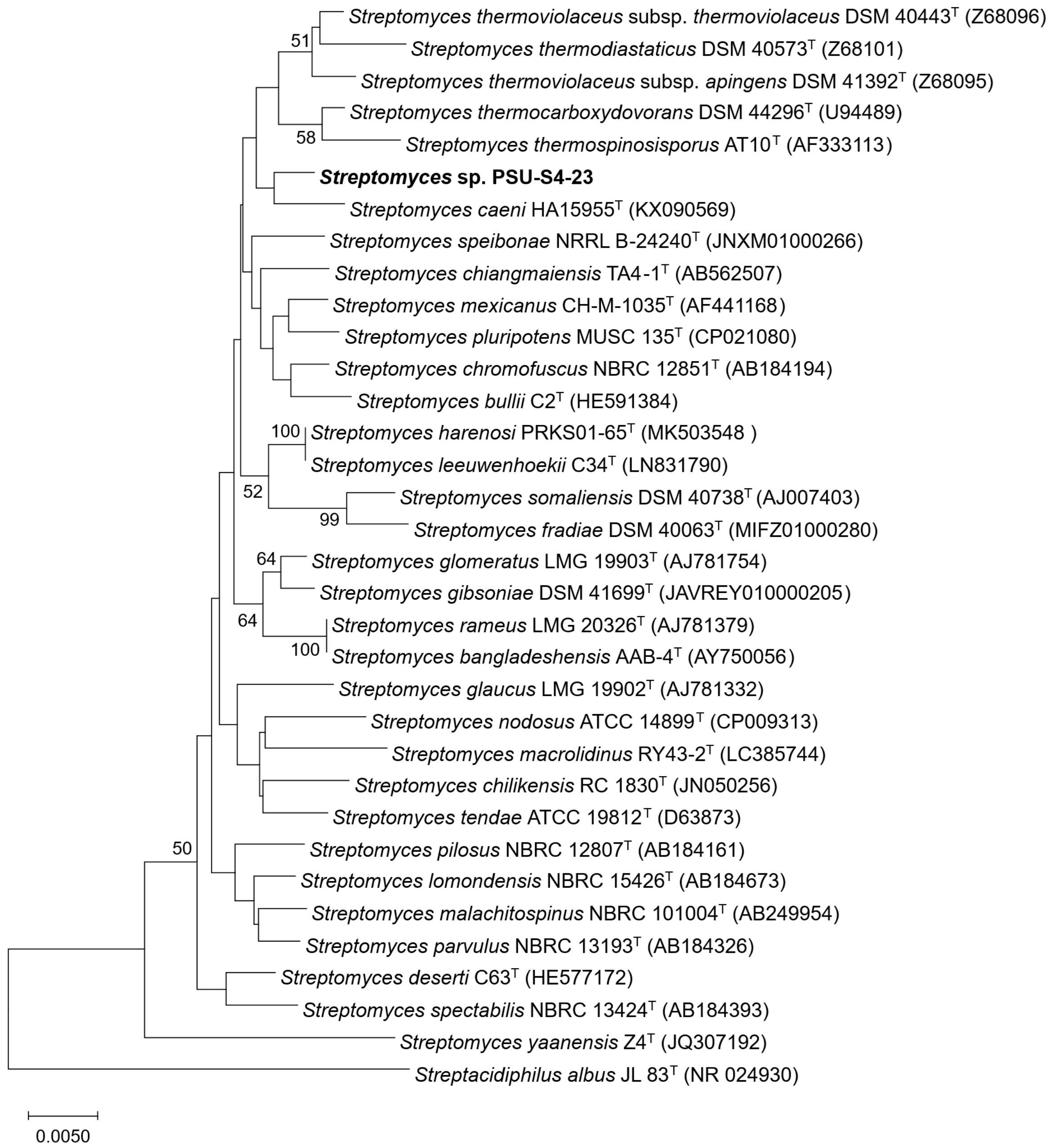

Figure S1. Neighbour-joining tree based on 16S rRNA gene sequences showing the relationship between strain PSU-S4-23 and other closely related *Streptomyces*. All positions containing gaps and missing data were eliminated. There were a total of 1389 positions in the final dataset. Bootstrap values (>50 %) based on 1000 replicates are shown at branch nodes. *Streptacidiphilus albus* JL 83^{T} was used as the outgroup. Bar, 0.005 nucleotide substitution rate (Knuc) units.

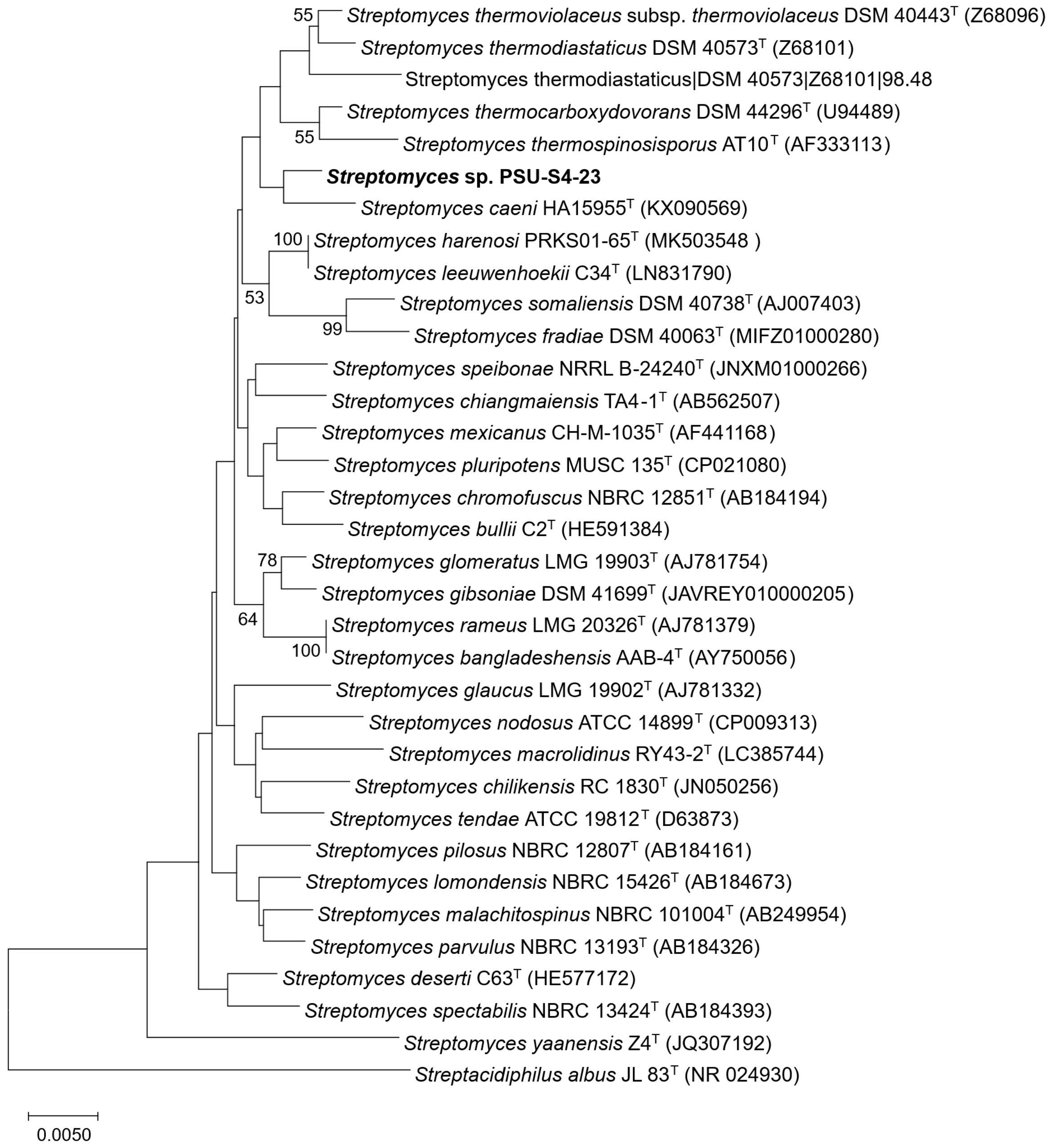

Figure S2. Maximum parsimony phylogenetic tree based on 16S rRNA gene sequences showing the relationship between strain PSU-S4-23 and closely related *Streptomyces*. All positions containing gaps and missing data were eliminated. There were a total of 1389 positions in the final dataset. *Streptacidiphilus albus* JL 83^T was used as an outgroup. Bootstrap values 50 % (based on 1000 replications) are shown at branch points. Bar, 0.005 substitutions per nucleotide position.

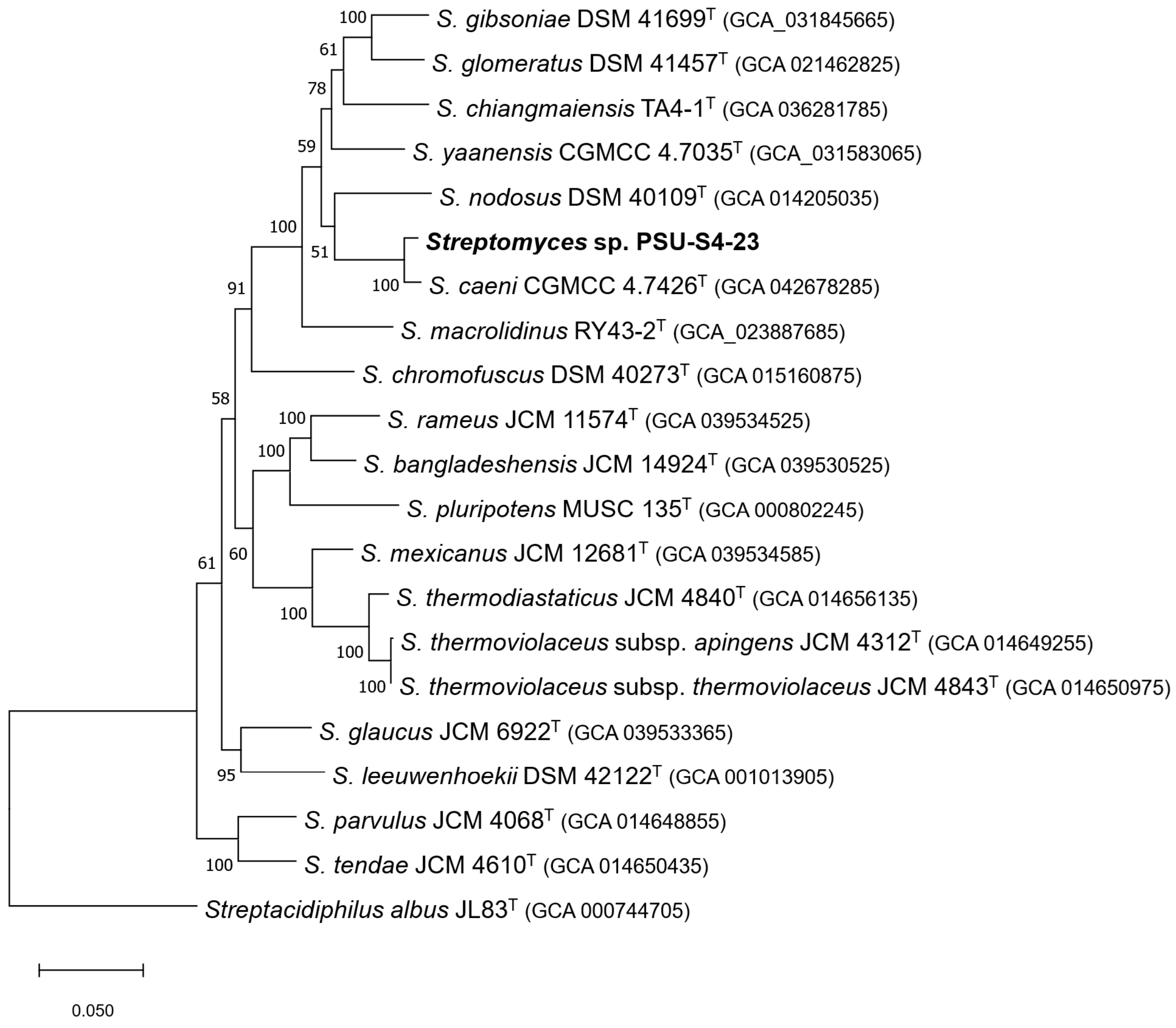

Figure S3. Phylogenomic tree generated by autoMLST showing the relationship of strain PSU-S4-23 to related species within the genus *Streptomyces*.

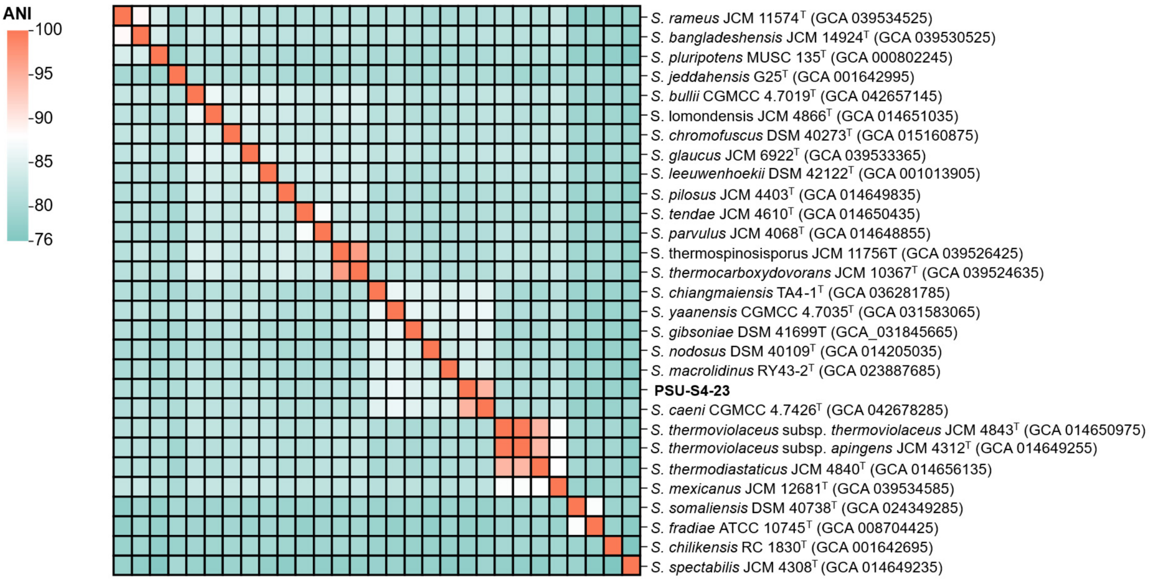

Figure S4. Average Nucleotide Identity (ANI) heatmap and clustering of *Streptomyces* genomes.

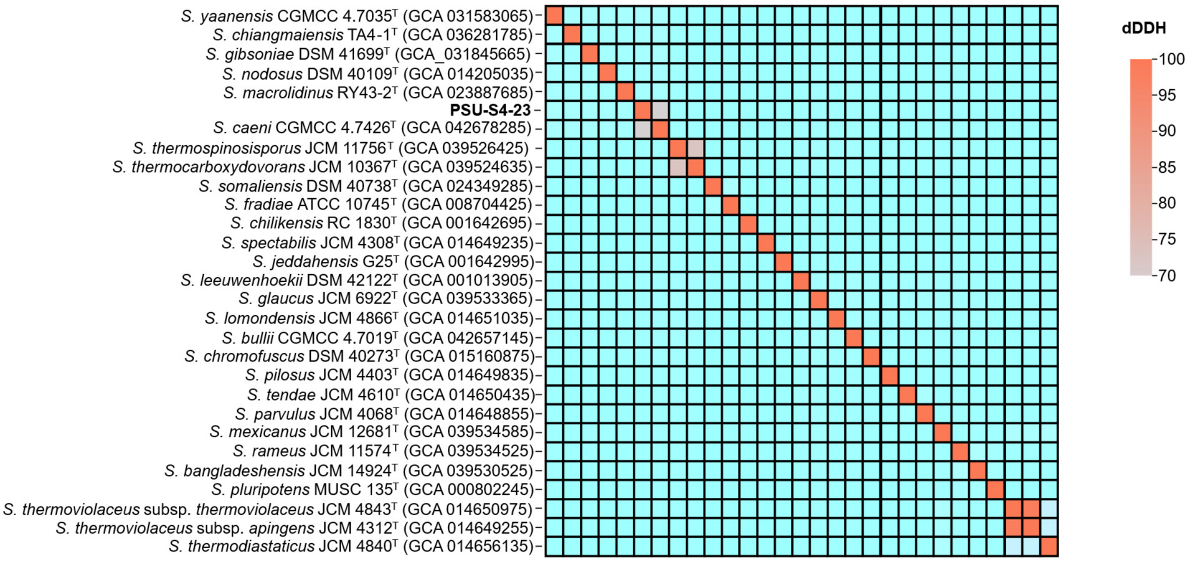

Figure S5. Digital DNA–DNA hybridization (dDDH) heatmap among *Streptomyces* genomes.

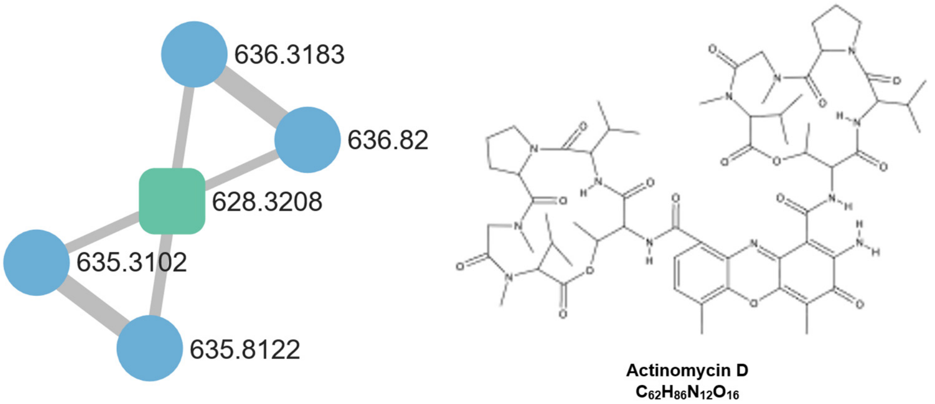

Figure S6. Actinomycin D–associated cluster in the GNPS molecular network of the ethyl-acetate extract from *Streptomyces* sp. PSU-S4-23. Each node represents m/z value of the parent ion and edge thickness signifies cosine score similarity. Each node represents the m/z value of a parent ion $[M+2H]^{2+}$, and edge thickness signifies the cosine similarity score between MS/MS spectra.

The molecular networking job can be publicly accessed at
https://gnps.ucsd.edu/ProteoSAFe/status.jsp?task=f70e25f4fc3f4f149d6037888f89f7be

Table S1. Assembly statistics and quality metrics for the draft genome of *Streptomyces* sp. PSU-S4-23.

Category	Metric	Value
Assembly (QUAST)	Number of contigs (≥ 0 bp)	3
	Total length	8,857,303 bp
	Largest contig	8,704,871 bp
	GC content	71.76 %
	N50	8,704,871 bp
	N90	8,704,871 bp
	auN	8,556,421.4 bp
	L50	1
	L90	1
	N's per 100 kbp	0.00 (No gaps)
Genome completeness		
BUSCO	BUSCO version	5.8.0
	Lineage dataset	Streptomycetales_odb10
	Complete BUSCOs (C)	99.7 % (1,574/1,579)
	Single-copy BUSCOs (S)	99.4 % (1,569)
	Duplicated BUSCOs (D)	0.3 % (5)
	Fragmented BUSCOs (F)	0.0 % (0)
	Missing BUSCOs (M)	0.3 % (5)
CheckM	CheckM version	1.0.18
	Marker lineage	Streptomycetaceae
	Completeness	100.0 %
	Contamination	2.22 %

Table S2. Comparative analysis of the BGC Cluster 20 in PSU-S4-23 and the actinomycin D cluster (BGC0000296).

PSU-S4-23 BGC cluster 20					Actinomycin D BGC (BGC0000296)						Amino-acid identity (%)
locus	start	end	strand	Amino-acid length	locus	start	end	strand	Amino-acid length	BGC product	
**ctg2_6390**	5573	5920	+	115	orf120	47456	47818	-	120	transposase	35.8
**ctg2_6398**	14798	15289	-	163	orf157	47962	48435	-	157	transposase	37.4
**ctg2_6401**	16299	16931	-	210	acmT	11598	12233	-	211	hypothetical protein	73.5
**ctg2_6402**	16928	17491	-	187	acmS	12244	12804	-	186	unknown	73.8
**ctg2_6403**	17501	17701	-	66	acmR	12817	13017	-	66	MbtH-like protein	78.8
**ctg2_6404**	17747	17983	-	78	acmD	13084	13320	-	78	4-MHA carrier protein	71.8
**ctg2_6405**	17980	19383	-	467	acmA	13317	14735	-	472	peptide synthetase	72.7
**ctg2_6406**	20001	27827	+	2608	acmB	15166	23001	+	2611	peptide synthetase	73.6
**ctg2_6407**	27824	40537	+	4237	acmC	22998	35741	+	4247	peptide synthetase	77.7
**ctg2_6408**	40609	41238	+	209	acmE	35738	36445	+	235	hypothetical protein	70.2
**ctg2_6409**	41243	42136	+	297	acmF	36480	37394	+	304	aryl formamidase	79.3
**ctg2_6410**	42133	42975	+	280	acmG	37391	38233	+	280	tryptophan 2,3-dioxygenase	77.1
**ctg2_6411**	43048	44310	+	420	acmH	38294	39556	+	420	kynureninase	81.4
**ctg2_6412**	44363	45403	+	346	acmL	9455	10495	-	346	methyltransferase	97.4
**ctg2_6415**	47031	47678	-	215	acmO	7181	7828	+	215	LbmU-like protein	89.8
**ctg2_6416**	47905	48747	-	280	acmP	6112	6954	+	280	TetR family transcriptional regulator	93.2
**ctg2_6417**	48863	49747	+	294	acmQ	5113	5997	-	294	siderophore-interacting protein	96.6
**ctg2_6418**	49734	50810	+	358	acmrA	4050	5033	-	327	ABC transporter ATPase subunit	88.0
**ctg2_6419**	50800	51567	+	255	acmrB	3293	4060	-	255	ABC 2-type transporter	97.3
**ctg2_6420**	51654	53924	+	756	acmrC	936	3206	-	756	UrvA-like protein	96.6
